# Chlorogenic Acid–Strontium-Containing Dual-Functional Bioresorbable External Stent Suppresses Venous Graft Restenosis via Hippo-YAP Signaling Pathway

**DOI:** 10.3390/jfb16070259

**Published:** 2025-07-11

**Authors:** Ge Zhu, Su Wang, Zhang Liu, Shengji Gu, Feng Chen, Wangfu Zang

**Affiliations:** 1 Department of Cardio-Thoracic Surgery, Shanghai Tenth People’s Hospital, School of Medicine, Tongji University, Shanghai 200072, China; zg01k39@rjh.com.cn (G.Z.); ws7202@shtrhospital.com (S.W.); 1910858@tongji.edu.cn (Z.L.); 2022110739@stu.njmu.edu.cn (S.G.); 2 Shanghai Key Laboratory of Craniomaxillofacial Development and Diseases, Shanghai Stomatological Hospital & School of Stomatology, Fudan University, Shanghai 201102, China

**Keywords:** vein graft failure, external stent, chlorogenic acid, neointimal hyperplasia, hippo signaling pathway

## Abstract

Vein graft restenosis remains a major complication following coronary artery bypass grafting (CABG), mainly due to the abnormal proliferation of vascular smooth muscle cells (VSMCs) and impaired endothelial repair. While external stents (eStents) can provide mechanical support and limit adverse remodeling, traditional metallic stents are non-degradable and may induce chronic inflammation and fibrosis. In contrast, many bioresorbable materials degrade too quickly or lack mechanical strength. These challenges highlight the need for external stents that combine sufficient mechanical strength with biodegradability to support long-term graft patency. This is the first study that develops a chlorogenic acid–strontium (SrCA)-loaded polycaprolactone bioresorbable eStent that inhibits VSMC proliferation and enhances endothelial repair via Hippo–Yes-associated protein (YAP) signaling, addressing vein graft restenosis post-CABG. Combining mechanical support and biodegradability, it overcomes the limitations of non-degradable stents and rapidly degrading biomaterials, elucidates the potential of natural polyphenol–metal ion complexes in vascular remodeling, and offers an innovative strategy for the prevention of vein graft restenosis.

## 1. Introduction

Coronary artery disease (CAD) remains a leading global health burden [1,2]. Coronary artery bypass grafting (CABG) is the gold standard treatment for multivessel disease and complex or severe anatomical stenoses [3,4], with saphenous vein grafts (SVGs) widely used due to their availability and length [5,6]. However, vein graft failure—primarily driven by restenosis from intimal hyperplasia—significantly compromises long-term outcomes [7]. This pathological remodeling, exacerbated by arterial pressure and cyclic stretch, leads to uncontrolled vascular smooth muscle cell (VSMC) proliferation and luminal narrowing [8,9]. Effective strategies to prevent vein graft restenosis are urgently needed to improve graft durability and clinical prognosis after CABG.

Several therapeutic strategies have been developed to mitigate vein graft remodeling, including gene therapy, modifications in vein harvesting techniques, and the use of external vein graft stents. Among these, external vein graft stents have demonstrated promising outcomes in enhancing graft patency rates and suppressing intimal hyperplasia [10,11,12,13]. Several studies have highlighted that the efficacy of stents largely depends on the materials and structural design used in their fabrication. For instance, Murphy et al. reported that polyester-based stents could contribute to vein graft failure, owing to their stiffness and incomplete conduit design [14]. The prolonged presence of foreign materials significantly heightens the risk of infection and immune rejection [15]. Consequently, bare-metal stents alone have shown limited efficacy in preventing vein graft failure [16,17]. To tackle these difficulties, the use of biodegradable materials has been suggested as a promising alternative. Polycaprolactone (PCL), a biodegradable and biocompatible polymer, is widely employed in tissue engineering, owing to its advantageous characteristics. Extrusion-based 3D printing has emerged as a promising method for fabricating customized stents tailored to the needs of personalized medicine [18,19].

The exact mechanisms by which external stents suppress intimal hyperplasia remain poorly understood. Recent studies have identified Yes-associated protein (YAP), a downstream effector of the Hippo signaling pathway, as a key player in vascular remodeling [20]. Additionally, YAP expression has been strongly linked to VSMCs proliferation and migration, key contributors to intimal hyperplasia and graft restenosis [21,22]. These insights suggest that modulating the Hippo-YAP axis may provide a novel strategy to mitigate pathological remodeling in vein grafts.

In recent years, biomaterials have garnered significant attention in medical research. Chlorogenic acid (CGA), for instance, has demonstrated promising effects, such as inhibiting platelet aggregation [23], reducing heart failure [24], and preventing myocardial infarction [25,26,27]. However, its low absolute bioavailability significantly limits its clinical utility. Incorporating strontium ions (Sr^2+^) into biomaterials has emerged as an effective strategy to enhance the biological performance of implants [28]. Notably, recent findings suggest that Sr^2+^ may protect myocardial tissue from ischemia/reperfusion injury [29]. These insights highlight the potential for novel biomaterial designs in addressing challenges related to vein grafts and cardiovascular health.

In this study, we developed a novel chlorogenic acid–strontium (SrCA) dual-functional bioresorbable eStent using electrospinning technology to fabricate custom-woven PCL tubular stents with optimized radial strength, axial flexibility, and luminal patency. These mechanical properties ensure adequate early-stage support while reducing long-term foreign body reactions during biodegradation [30]. To our knowledge, this is the first SrCA-loaded eStent designed to inhibit VSMC proliferation and promote endothelial repair via modulation of the Hippo-YAP signaling pathway, thereby targeting the pathological mechanisms underlying vein graft restenosis after CABG. Our findings not only elucidate the mechanistic role of this biofunctional eStent in suppressing vein graft remodeling but also highlight its clinical potential as a next-generation therapeutic strategy for improving long-term graft patency in vascular surgery.

## 2. Materials and Methods

### 2.1. SrCA Fabrication

**Figure 1:**
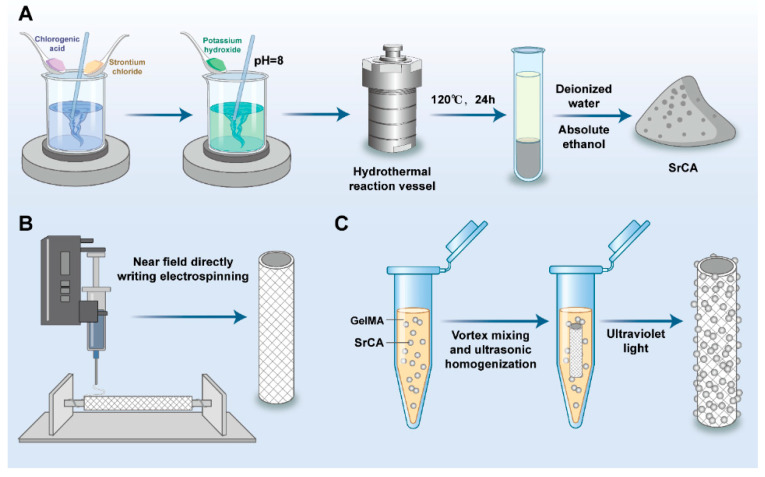
Schematic representation of the fabrication of (**A**) SrCA, (**B**) PCL stent, and (**C**) SrCA-loaded eStent.

The preparation method for SrCA is as follows (Figure 1A): Dissolve 26 mg of CGA and 10 mg of strontium chloride in 15 mL of deionized water under magnetic stirring for 10 min until fully dissolved. Add 5 mg of potassium hydroxide to the mixed solution, adjusting the pH to 8. Transfer the solution into a 25 mL polytetrafluoroethylene (PTFE) hydrothermal reaction vessel using a pipette. Place the vessel in a preheated oven at 120 °C and allow the reaction to proceed for 24 h. After completion, cool the reaction mixture naturally to room temperature. Collect the reaction product via centrifugation and wash it 3–5 times with deionized water and absolute ethanol, respectively. Finally, disperse the product in absolute ethanol and store it at 4 °C for future use.

### 2.2. SrCA eStent Fabrication

The preparation method for the PCL stent is as follows (Figure 1B): Dissolve 3.5 g of PCL in 10 mL of trifluoroethanol under magnetic stirring in a 50 °C thermostatic water bath for 12 h to ensure complete dissolution, forming a 20% (*w*/*w*) PCL solution. Load the prepared PCL solution into a 10 mL (21-gauge) syringe and employ a near-field directly writing electrospinning platform (NFDWE) (Changsha Nayi Instrument Technology Co., Changsha, China) for stent fabrication. During the process, set the electrostatic direct writing rate at 0.1 mL/h, maintain a voltage of 2.5 kV, adjust the height to 3 mm, and control the collection speed at 10 mm/s. The resulting PCL stent is maintained at room temperature for future application.

The preparation method for the porous gelatin methacryloyl (GelMA) hydrogel loaded with SrCA is as follows (Figure 1C): Add an appropriate amount of phosphate-buffered saline (PBS) to a bottle containing porous GelMA to prepare a 6% (*w*/*v*) GelMA hydrogel precursor solution. Stir the prepared solution in a 37 °C light-protected water bath using a magnetic stirrer for 1 h. Immediately filter the solution utilizing a 0.22 μm sterile needle filter to ensure sterility and set it aside for further use. Weigh pre-prepared SrCA using centrifugation and mix it with the porous GelMA precursor solution in a 37 °C thermostatic water bath. Prepare mixtures at concentrations of 20% (*w*/*v*), 10% (*w*/*v*), 5% (*w*/*v*), and 2.5% (*w*/*v*) by weight. Use vortex mixing and ultrasonic homogenization to ensure uniform distribution. Additionally, prepare a blank GelMA group with no additives. Transfer the SrCA /GelMA mixtures into four separate EP tubes. Submerge the pre-fabricated PCL stents into these solutions. After immersion, remove the stents and expose them to ultraviolet light for 3 min at room temperature in the absence of light to induce crosslinking and self-curing. The final products include GelMA-coated eStents with SrCA concentrations of 20% (*w*/*v*), 10% (*w*/*v*), 5% (*w*/*v*), and 2.5% (*w*/*v*), as well as a blank GelMA hydrogel eStent for control experiments.

### 2.3. Characterization of SrCA and SrCA eStents

The morphology and surface structure of SrCA and the eStents were examined using scanning electron microscopy (SEM, ZEISS, Gemini 300, Oberkochen, Germany) and transmission electron microscopy (TEM, FEI, Tecnai G2 F20, Hillsboro, OR, USA). TEM was specifically employed to analyze the size and morphology of SrCA. Selected area electron diffraction (SAED, FEI, Tecnai G2 F20, Hillsboro, OR, USA) was performed to determine whether SrCA exhibits a crystalline structure. Energy-dispersive X-ray spectroscopy (EDS, Xplore, Oxford, UK) elemental mapping was conducted to analyze the elemental composition of SrCA and eStents. Fourier-transform infrared spectroscopy (FTIR, Thermo Fisher, Waltham, MA, USA) was used to assess SrCA chemical properties. The tensile stress–strain mechanical performance of the eStents was evaluated using an electronic tensile testing machine (HY-940FS, Shanghai, China).

### 2.4. Cell Culture and SrCA eStent Extract Preparation

Rat aortic smooth muscle cells (A7r5, ATCC^®^ CRL-1444^TM^) were cultured in Dulbecco’s Modified Eagle Medium (DMEM, Gibco, Waltham, MA, USA) supplemented with 10% fetal bovine serum (FBS, Gibco), 100 U/mL penicillin, and 100 μg/mL streptomycin. Cells were maintained at 37 °C in a humidified 5% CO_2_ atmosphere and passaged at 80–90% confluence using 0.25% trypsin–EDTA (Gibco).

SrCA eStents were sterilized under UV light (254 nm, 30 min per side) and pre-conditioned in serum-free DMEM for 24 h at 37 °C. SrCA eStent extracts were collected by incubating SrCA eStents in serum-free medium (1 cm^2^ stent/mL medium) for 72 h.

### 2.5. Cell Counting Kit-8 (CCK-8) Assay

The CCK-8 assay was conducted in accordance with the manufacturer’s guidelines to evaluate the cytotoxicity of SrCA eStents on A7r5 cells. A7r5 cells were plated into 96-well plates at a density of 2000 cells per well and maintained on tissue culture plates (TCPs) for 24 and 48 h with various concentrations of SrCA eStent extract. Subsequently, 10 μL of CCK-8 reagent was added to each well, followed by a two-hour incubation in a cell culture incubator. Finally, the absorbance of each well was measured at 450 nm with a microplate reader (Bio Tek Synergyz, Winooski, VT, USA).

### 2.6. Live/Dead Assay

Cells were seeded onto 24-well plates containing SrCA eStent extracts at a density of 5 × 10^4^ cells/cm^2^ and cultured for 72 h. Cells were washed twice with PBS and incubated with 2 μM Calcein-AM (live cells, green fluorescence) and 4 μM propidium iodide (PI, dead cells, red fluorescence) (Thermo Fisher, Waltham, MA, USA) in PBS for 30 min at 37 °C. Fluorescent images were obtained using a confocal laser scanning microscope (LEICA DMI6000B, Wetzlar, Germany).

### 2.7. Cell Migration Assay

Cells were seeded in 6-well plates (2 × 10^5^ cells/well) and grown to 100% confluence. A standardized scratch was created using a 200 μL sterile pipette tip. Debris was removed by washing with PBS. Cells were treated with (a) SrCA eStent extracts, (b) SrCA-free eStent extracts, or (c) DMEM (blank control group). Scratch closure was monitored at 0, 24, and 48 h using an inverted phase-contrast microscope (Olympus CKX31SF, Tokyo, Japan). ImageJ software (version 1.52v) was adopted for the quantitative analysis of the cell migration amount.

### 2.8. Quantitative Real-Time PCR

Total RNA was extracted using TRIzol^®^. YAP/TAZ target genes (CTGF, CYR61) and contractile markers (α-SMA, SM22α) were amplified with SYBR Green primers (sequences provided in Table S1).

As per the manufacturer’s guidelines, total RNA was extracted from A7r5 cells co-cultured with the SrCA eStent extract using TRIzol reagent (Shanghai Shenggong Biotechnology Co., Shanghai, China). Complementary DNA (cDNA) was synthesized using the PrimeScript^TM^ RT Reagent Kit with gDNA Eraser (TaKaRa, Kusatsu, Japan). Quantitative real-time PCR (RT-qPCR) was performed using the TB Green Premix Ex Taq^TM^ II kit (TaKaRa, Kusatsu, Japan) on a fluorescence real-time PCR system (CFX96^TM^, Bio-Rad, Hercules, CA, USA), with GAPDH used as the internal control. Gene expression levels were determined utilizing the 2^−ΔΔCt^ method. Primer sequences are provided in Supplementary Materials Table S1.

### 2.9. Western Blot

The proteins from A7r5 co-cultured with SrCA eStent extract were extracted utilizing RIPA lysis buffer (Gibco, Waltham, MA, USA), which included a combination of protease and phosphatase inhibitors. Equal quantities of protein were separated by 10% sodium dodecyl–sulfate polyacrylamide gel electrophoresis (SDS-PAGE) and subsequently transferred onto polyvinylidene difluoride (PVDF) membranes (Beyotime, Shanghai, China). After blocking, the membranes were incubated with a primary antibody against YAP (Absin, Shanghai, China), followed by incubation performed with the relevant HRP-conjugated secondary antibody. The expression level of glyceraldehyde 3-phosphate dehydrogenase (GAPDH) served as an internal control.

### 2.10. Surgical Procedure

This research and all animal procedures received approval from the Ethics Committee of Shanghai Tenth People’s Hospital, Tongji University School of Medicine, and adhered to the National Institutes of Health Guide for the Care and Use of Laboratory Animals (NIH Publications No. 85-23 [31], revised 1996).

Sprague–Dawley rats (male, 300–350 g, *n* = 6/group) were anesthetized via intraperitoneal injection of 3% pentobarbital sodium at a dosage of 0.1 mL/100 g. The right external jugular vein was meticulously isolated, dissected, and immersed in a heparin saline solution. Following the immobilization and transection of the ipsilateral common carotid artery, a vein graft was implanted utilizing the “cuff” technique. Both ends of the dissected artery were everted over a custom-made cuff (1.5 mm), created from the cannula of a 20G indwelling needle and secured using 8-0 Prolene sutures. The cuffed artery was then inserted into the harvested jugular vein. Prior to ligation at the anastomotic junction, the grafts in the stent group were externally wrapped with SrCA eStents matching the vein length. Non-stented vein grafts served as controls. Animals in each group were euthanized at 2 and 4 weeks post-implantation before vein graft harvesting.

### 2.11. Histomorphology

The harvested vein grafts underwent fixation in 4% paraformaldehyde, were embedded in paraffin, and subsequently sectioned into 5 μm thick slices. Hematoxylin and eosin (H&E) staining was performed following standard protocols to characterize morphological changes. To identify the smooth muscle layer surrounding neointimal hyperplasia, elastic van Gieson (EVG) staining was conducted according to the manufacturer’s instructions. A quantitative evaluation of vessel wall thickness was performed using ImageJ software (version 1.52v).

### 2.12. Statistical Analysis

Data were analyzed with SPSS v22.0 software (IBM, Armonk, NY, USA). A one-way analysis of variance was employed to assess the differences among groups. An unpaired Student’s *t*-test was employed to evaluate the differences between the two groups. All measurements were performed three times independently, and the results are expressed as the mean ± SD. *p* < 0.05 was considered significant.

## 3. Results

### 3.1. Fabrication and Characterization of SrCA

**Figure 2:**
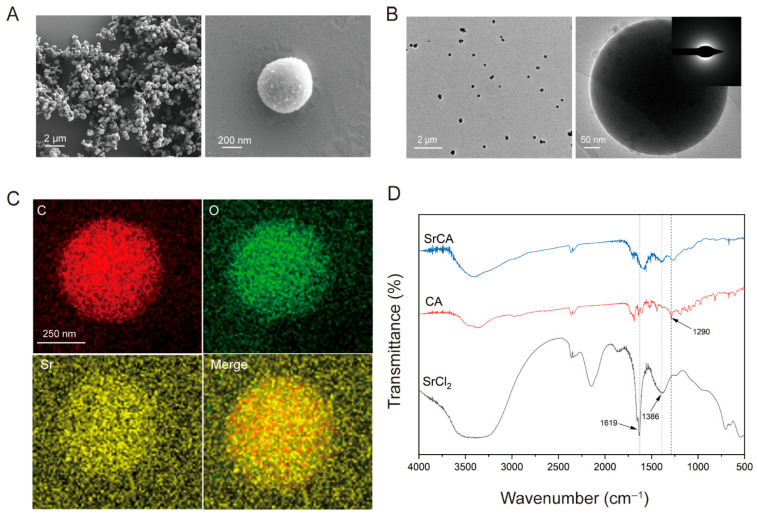
(**A**) SEM, (**B**) TEM, and SAED. (**C**) Elemental mappings of SrCA. (**D**) FTIR of SrCA, CA, and SrCl_2_.

The SrCA complex (Figure 2A) displayed uniform spherical morphology (avg. 500 nm, Figure S1) with smooth surfaces (Figure 2B). SAED patterns (Figure 2B inset) displayed diffuse rings, indicating the amorphous nature of the SrCA. EDS elemental mapping (Figure 2C) demonstrated a uniform spatial distribution of carbon (C), oxygen (O), and strontium (Sr) within the composite. FTIR spectroscopy (Figure 2D) identified characteristic absorption bands, with 1619 cm^−1^ and 1386 cm^−1^ corresponding to Sr-Cl vibrational modes from the strontium chloride precursor and 1290 cm^−1^ attributable to aromatic C-C skeletal vibrations of CGA. The presence of these distinct peaks in the SrCA spectrum, along with the absence of isolated precursor signatures, confirmed successful coordination between CGA and strontium ions through hydrothermal synthesis.

### 3.2. Fabrication and Characterization of SrCA eStents

**Figure 3:**
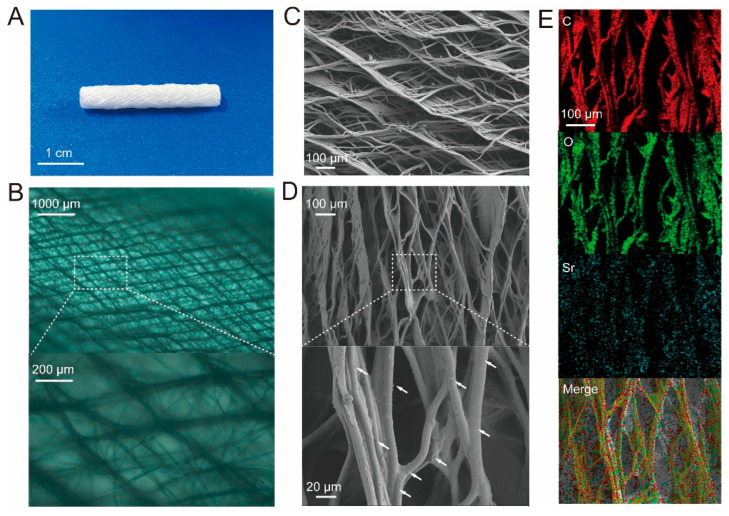
(**A**,**B**) Optical photograph; (**C**) SEM PCL eStent. (**D**) SEM. (**E**) Elemental mappings of SrCA eStent. White arrows refer to SrCA nanoparticles.

The PCL stent exhibited a 3D porous network (Figure 3A–C) with smooth fibers. Following the GelMA photocrossloading of SrCA nanoparticles onto the stents, SEM imaging (Figure 3D) showed retained three-dimensional porosity with distinct surface roughening. SrCA nanoparticles (indicated by white arrows) were uniformly distributed along the fiber surfaces. EDS elemental mapping (Figure 3E) verified a homogeneous dispersion of carbon (C), oxygen (O), and strontium (Sr) across the fibrous network, confirming successful SrCA immobilization. Mechanical tests demonstrated that the stents have excellent radial ductility and cyclic resilience (Figure S2).

### 3.3. eStent Inhibited Intimal Hyperplasia of Vein Grafts and Regulated Vascular Remodeling

**Figure 4:**
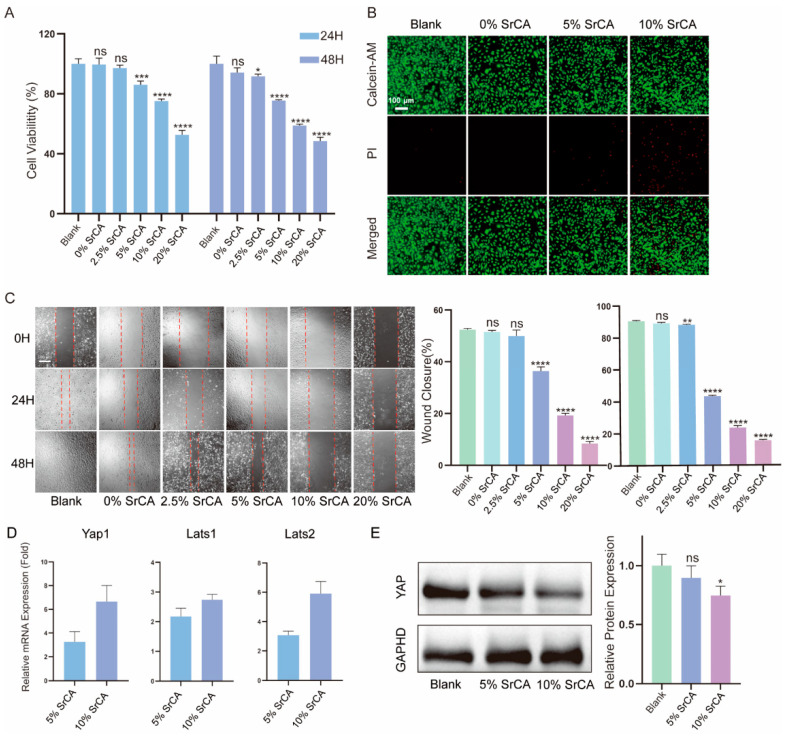
(**A**) CCK-8 assay results of VSMCs after co-culture with different concentrations of SrCA eStent extracts for 24 and 48 h. (**B**) LIVE/DEAD assay results of VSMCs after co-culturing with different concentrations of SrCA eStent extracts for 24 h. (**C**) Cell migration assay results of VSMCs after co-culturing with different concentrations of SrCA eStent extracts for 24 and 48 h, and a statistical diagram of the corresponding wound healing rate percentage. (**D**) RT-qPCR results of VSMCs after co-culturing with different concentrations of SrCA eStent extracts for 24 h. (**E**) Western blot analysis of VSMCs after co-culturing with different concentrations of SrCA eStent extracts for 24 h. (ns indicates not significant, * *p* < 0.05, ** *p* < 0.01, *** *p* < 0.001, **** *p* < 0.0001. Green fluorescence: Calcein-AM represents live cells; red fluorescence: PI represents dead cells).

The CCK-8 assay (Figure 4A) demonstrated a concentration-dependent inhibition of VSMC proliferation by the eStents. At 48 h, viability decreased to 75.55% (5% SrCA), 58.86% (10% SrCA), and 48.50% (20% SrCA) compared to the blank group (*p* < 0.0001). Notably, 0% of the SrCA stents exhibited no cytotoxicity (94.14% viability), confirming the biocompatibility of GelMA/PCL composites. Conversely, 20% SrCA significantly reduced VEC viability (63.39%, Figure S3).

The LIVE/DEAD assay results (Figure 4B) demonstrated that both 5% and 10% SrCA effectively inhibited the growth of VSMCs. A cell migration assay (Figure 4C) revealed a dose-dependent suppression of VSMC migration. Wound closure rates at 48 h decreased from 90.50% (blank) to 43.22% (5% SrCA) and 23.45% (10% SrCA) (*p* < 0.0001).

Molecular analyses demonstrated SrCA-mediated Hippo pathway activation. RT-qPCR (Figure 4D) showed an upregulation of Yap1 (6.66-fold), Lats1 (2.74-fold), and Lats2 (5.91-fold) in 10% SrCA-treated VSMCs (*p* < 0.001 vs. blank). Western blotting (Figure 4E) confirmed corresponding YAP downregulation, indicating a pathway-specific suppression of VSMC proliferation.

### 3.4. In Vivo Efficacy of EStents in Vein Grafts

**Figure 5:**
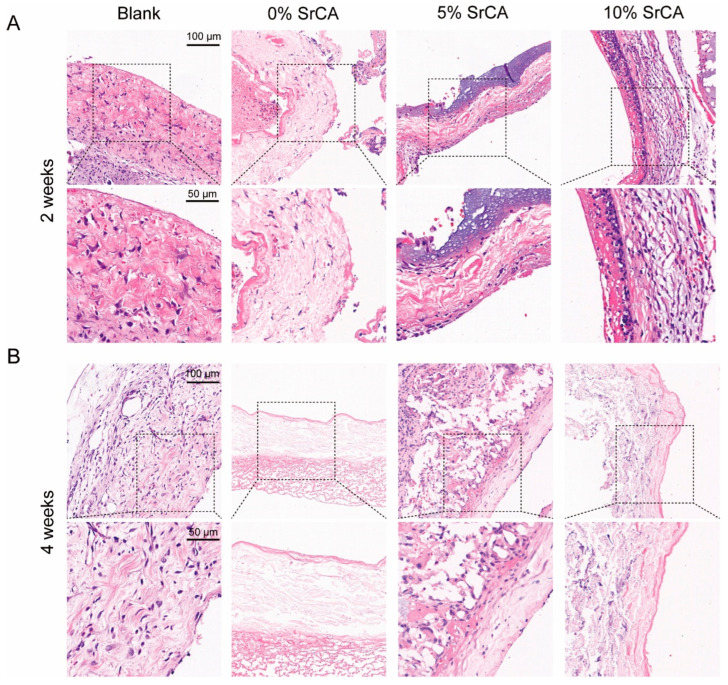
H&E staining results of vein graft samples at (**A**) 2 weeks and (**B**) 4 weeks after placement of the SrCA eStents.

**Figure 6:**
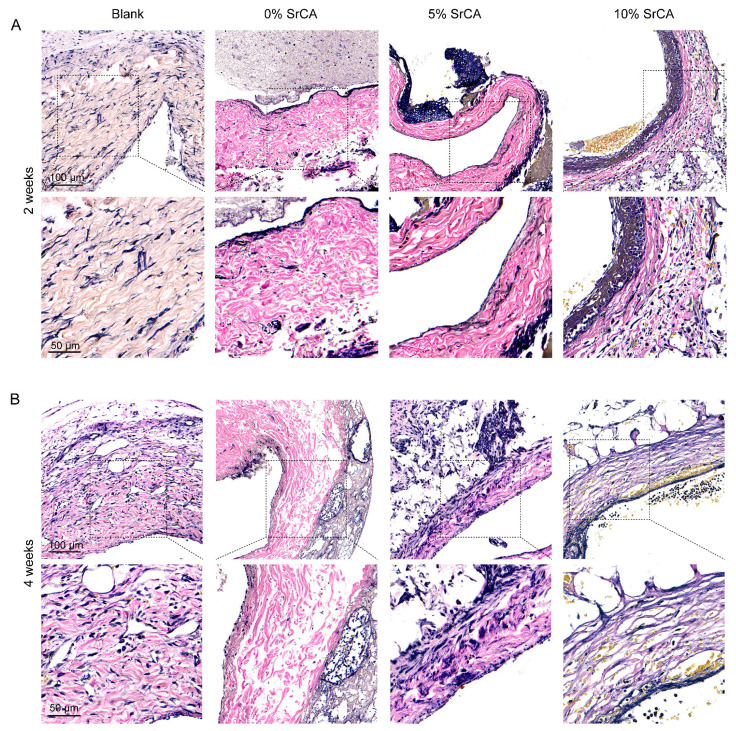

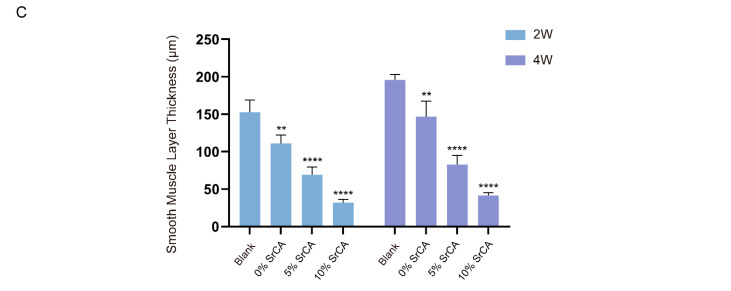
EVG staining results of vein graft samples at (**A**) 2 weeks and (**B**) 4 weeks after placement of the SrCA eStents. (**C**) Statistical diagram of smooth muscle layer thickness. (** *p* < 0.01, **** *p* < 0.0001).

H&E staining (Figure 5) demonstrated that the stent-implanted groups (5% and 10% SrCA) maintained endothelial integrity at both 2-week and 4-week time points, with continuous and intact luminal surfaces. EVG staining (Figure 6) further revealed a significant reduction in smooth muscle layer thickness in the stent groups. At 4 weeks post-implantation, the average smooth muscle layer thickness in the 5% SrCA and 10% SrCA groups decreased to 82.87 μm and 41.61 μm, respectively, compared to 195.77 μm in the blank group (*p* < 0.0001) (Figure 6C). These findings indicate that SrCA eStents effectively suppress neointimal hyperplasia and vascular remodeling in vivo.

## 4. Discussions

CABG remains a primary treatment strategy for severe CAD [32,33,34]. However, the long-term efficacy of CABG is notably constrained by vein graft restenosis [6]. When exposed to the arterial high-pressure environment, vein grafts—characterized by thin vessel walls and a lack of elastic fibers—are prone to excessive dilation and endothelial injury. This initiates a cascade of pathological responses, including VSMC proliferation and migration, along with abnormal extracellular matrix (ECM) deposition, ultimately leading to and exacerbating neointimal hyperplasia (NIH) [8,9,35]. Therefore, minimizing mechanical stress is an effective strategy for enhancing vein graft patency. In recent years, external vascular stents have garnered significant attention due to their mechanical support properties. By limiting graft overexpansion and providing radial stabilization, eStents reduce wall stress and have been shown to lower the incidence of NIH [10,11,12]. However, purely mechanical intervention cannot block the molecular mechanisms driving VSMC proliferation. Moreover, traditional metallic eStents are non-degradable, often inducing chronic inflammation and fibrotic remodeling of the vessel wall [15,16]. On the other hand, many bioresorbable materials degrade too rapidly or lack sufficient mechanical strength to provide long-term support [36]. Against this backdrop, there is an urgent necessity to develop external stents that integrate mechanical adaptability, biodegradability, and targeted regulatory functions to address both biomechanical and biological challenges in vein graft failure.

PCL has been widely utilized in clinical applications, owing to its superior biocompatibility, stiffness, mechanical elasticity, biodegradability, non-toxicity, thermal stability, and its favorable rheological and viscoelastic properties [37]. Moreover, polycaprolactone (PCL) has been demonstrated to undergo complete metabolic degradation and excretion, with no associated systemic toxicity [38]. Importantly, its controlled degradation rate (approximately two years for complete resorption), low elastic modulus (300–400 MPa), and minimal inflammatory response make it an ideal candidate material for external vein graft stents [37,39]. Compared to the brittleness of polylactic acid (PLA) and the rapid degradation of polyglycolic acid (PGA), the gradual degradation profile of PCL enables a progressive transfer of mechanical load to the healing vessel, facilitating adaptive remodeling during the vascular recovery process [37]. In this study, a three-dimensional porous PCL stent was fabricated using electrostatic direct writing technology. The eStent’s architecture was designed not only to provide mechanical support but also to create a permissive microenvironment for cellular infiltration and vascular regeneration.

From a pharmacological perspective, although the local delivery of rapamycin has been shown to partially suppress VSMC activity, its non-selective cytotoxicity often results in delayed endothelial repair and late-stage thrombosis [40]. The synergistic effect of CGA and strontium ions (Sr^2+^) offers a novel strategy for the inhibition of NIH. As a natural polyphenol, CGA has been demonstrated to effectively counteract adrenaline-induced vascular injury by scavenging reactive oxygen species (ROS), inhibiting DNA damage signaling pathways, and maintaining VSMC homeostasis [41]. This highlights its potential as a natural therapeutic candidate for vascular diseases such as atherosclerosis. However, its application in vein grafts has not yet been reported. Strontium ions, on the other hand, exert pro-angiogenic effects by activating signaling pathways such as vascular endothelial growth factor (VEGF), significantly upregulating the expression of CD31 (an endothelial marker) and α-smooth muscle actin (α-SMA), thereby promoting neovascularization [42]. Additionally, Sr^2+^ modulates the local microenvironment and reduces the release of inflammatory cytokines [42]. Recent studies have shown that methacrylated gelatin (GelMA), which combines the advantages of natural materials and synthetic polymers, exhibits excellent biocompatibility while retaining bioactivity and possessing favorable mechanical properties [43]. The presence of matrix metalloproteinase (MMP)-sensitive sites and the arginine–glycine–aspartic acid (RGD) sequences in gelatin facilitates cell adhesion, migration, proliferation, and differentiation [43]. Furthermore, due to its highly porous structure, GelMA provides efficient channels for nutrient and waste exchange, as well as ample space for cell growth, significantly enhancing cellular proliferation. The RGD motifs in gelatin offer anchoring sites for cell adhesion, while Sr^2+^ activates intracellular signaling pathways to further support endothelial cell function [42]. This is the first study that reports the integration of CGA and Sr^2+^ into a composite complex (SrCA), which is incorporated into the PCL eStent via GelMA-based photocrosslinking, achieving dual-functional synergistic release.

The 10% SrCA group showed 58.9% VSMC proliferation inhibition (Figure 4A) and promoted endothelial cell growth (Figure S3). A scratch assay revealed 23.4% wound closure at 48h (Figure 4C), indicating the potent suppression of VSMC migration. The concentration-dependent dual effect of the SrCA eStent—suppressing VSMCs while supporting endothelial cells—highlights its therapeutic window advantage. Previous studies also support this conclusion, demonstrating that CGA possesses excellent biocompatibility [44,45]. Similarly, Sr^2+^ has been shown to exhibit favorable biocompatibility and low cytotoxicity [46,47]. This bifunctional property overcomes the limitations of conventional agents such as rapamycin, which inhibit neointimal hyperplasia but impair endothelial repair [48].

The Hippo signaling pathway is an evolutionarily conserved regulatory mechanism initially discovered in Drosophila, where it is essential for suppressing cell proliferation and controlling organ size [49,50]. In mammals, the core components of this pathway include the serine/threonine kinases Lats and their downstream effectors YAP (Yes-associated protein) and TAZ (transcriptional co-activator with PDZ-binding motif) [51]. When activated, YAP and TAZ translocate into the nucleus, where they associate with TEA domain (TEAD) family transcription factors, driving the expression of genes related to cell proliferation, apoptosis, and migration [51]. Conversely, phosphorylated YAP is rendered inactive and is retained in the cytoplasm, thereby exerting growth-suppressive effects. Recent studies have demonstrated a strong association between dysregulation of the Hippo pathway and cardiovascular diseases, particularly in the context of VSMC phenotypic switching and vascular remodeling [21]. Wang et al. reported that YAP overexpression promotes VSMC proliferation and migration, whereas genetic knockout or pharmacological inhibition of YAP—such as with verteporfin—significantly attenuates neointimal formation following vascular injury [52,53].

RT-qPCR analysis in this study revealed a concentration-dependent upregulation of Hippo pathway-related gene expression in VSMCs treated with the extract of the SrCA eStents. Compared with the blank control group, the mRNA expression levels of Yap1, Lats1, and Lats2 were elevated by 3.26-fold/6.66-fold, 2.18-fold/2.74-fold, and 3.07-fold/5.91-fold, respectively, in the 5% SrCA and 10% SrCA groups (Figure 4D). This finding was further validated at the protein level by Western blotting, which showed a dose-dependent decrease in nuclear YAP expression (Figure 4E). The inverse correlation between increased Yap1 mRNA levels and decreased nuclear YAP expression suggests that SrCA effectively inhibits downstream pro-proliferative signaling by enhancing LATS-mediated YAP phosphorylation and subsequent degradation.

A histopathological evaluation further confirmed the therapeutic efficacy of the SrCA eStents. H&E staining revealed well-preserved endothelial continuity in the eStent-implanted groups, with a smaller area of endothelial denudation compared to the blank control group (Figure 5). EVG staining showed that at 4 weeks post-implantation, neointimal thickness in the 5% SrCA and 10% SrCA groups was reduced relative to the control group (Figure 6). Importantly, the mechanical stability of the eStents, combined with the pharmacological effects of SrCA, provided a dual-mode intervention: mechanical support reduced vessel wall tension, while Hippo pathway activation suppressed VSMC proliferation and preserved endothelial function. Collectively, these effects contributed to a marked reduction in neointimal hyperplasia in the 10% SrCA group.

Although this study demonstrated the efficacy of the SrCA eStents in inhibiting venous graft restenosis, several limitations should be acknowledged. First, while rodent vein graft models are cost-effective and suitable for preliminary screening, they fail to fully replicate the hemodynamic stresses and chronic inflammatory responses seen in human CABG. Second, the 4-week observation period may be insufficient to evaluate long-term outcomes. Third, although our findings suggest that the activation of the Hippo-YAP/TAZ pathway is a key mechanism, proteomic or phosphoproteomic analyses were not conducted to comprehensively identify potential off-target effects or synergistic signaling networks. Fourth, we did not yet assess the systemic toxicity or detailed cellular responses in non-vascular healthy cells such as fibroblasts, which are essential for ensuring biocompatibility and biosafety. Lastly, clinical translation will require validation in large animal models, such as porcine coronary vein graft models. Addressing these limitations will strengthen the therapeutic rationale for the use of SrCA eStents in vein graft interventions.

## 5. Conclusions

In this study, a biodegradable eStent loaded with SrCA was successfully developed, offering an effective strategy to prevent vein graft restenosis through a unique “mechanical–chemical” synergistic mechanism. Experimental results demonstrated that the SrCA eStent significantly inhibited VSMC proliferation by activating the Hippo-YAP/TAZ signaling pathway while simultaneously promoting VEC growth. This dual functionality is attributed to the synergistic effects of CGA, known for its antioxidant and anti-inflammatory properties, and Sr^2+^, which enhances endothelial repair. Despite the limitations associated with rodent models and short-term observation, this study is the first to reveal the targeted regulatory potential of natural polyphenol–metal ion complexes in vascular remodeling, providing a theoretical foundation for the development of next-generation “pro-healing” vascular stents. In future studies, we plan to employ large animal models to further validate our findings and facilitate clinical translation.

## Data Availability

The original contributions presented in this study are included in the article and Supplementary Materials. Further inquiries can be directed to the corresponding author.

## Supplementary Materials

The following supporting information can be downloaded at: https://www.mdpi.com/article/10.3390/jfb16070259/s1, Figure S1: DLS Analysis of SrCA; Figure S2: Mechanical Properties of EStent; Figure S3: CCK-8 Assay of VECs; Table S1: Forward and reverse primer sequences used for each target gene synthesis in the RT-qPCR reaction.
