# Supplementary material for: Chlorogenic Acid–Strontium-Containing Dual-Functional Bioresorbable External Stent Suppresses Venous Graft Restenosis via Hippo-YAP Signaling Pathway
Source: J Funct Biomater. 2025 Jul 11;16(7):259. doi: 10.3390/jfb16070259 (PMC12295672; doi:10.3390/jfb16070259)
Supplement: Supplementary file 1 [file jfb-16-00259-s001.zip › jfb-3699812-supplementary.pdf]

Dual-Functional Bioresorbable External Stent with Chlorogenic Acid-Strontium Synergy Suppresses Venous Graft Restenosis via Hippo-YAP Signaling Pathway

1. DLS Analysis of SrCA

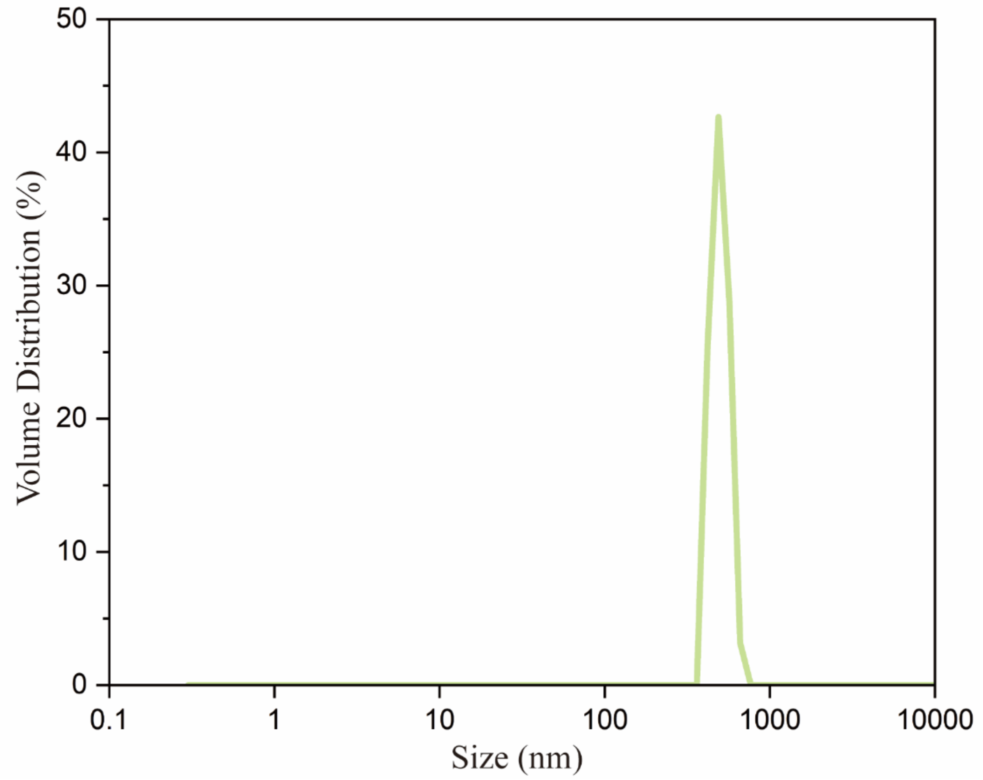

Figure S1. DLS analysis of SrCA.

2. Mechanical Properties of EStent.

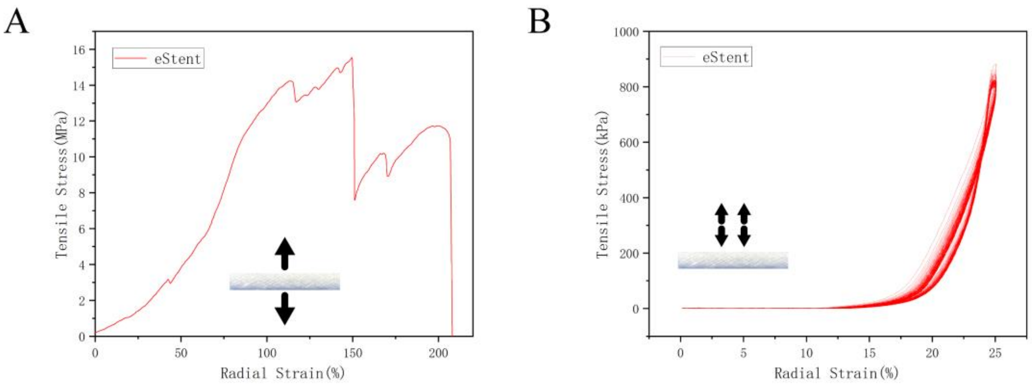

Figure S2. Mechanical properties of eStent. A. Ultimate radial tensile test; B. Cyclic radial

tensile test.

3. CCK-8 Assay of VECs

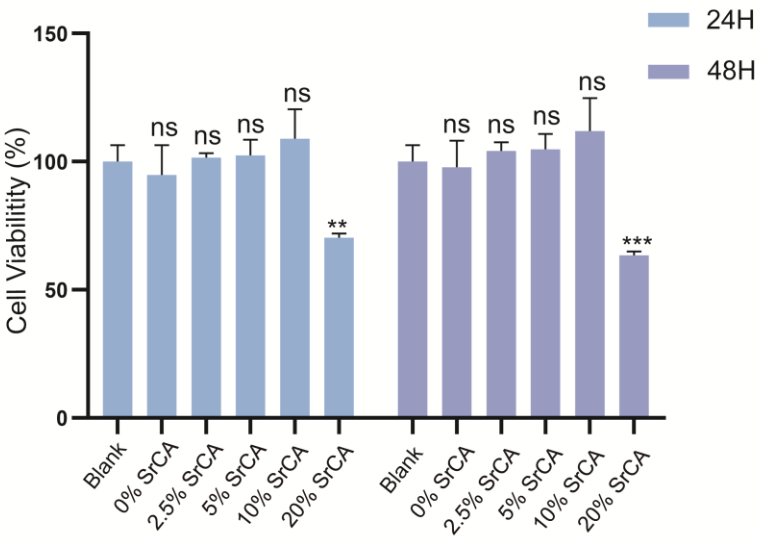

Figure S3. CCK-8 assay results of VECs after co-culture with different concentrations of SrCA eStents extract for 24 and 48 hours. (* P<0.05, ** P<0.01, *** P<0.001)

4. Sequences

Table S1 Forward and reverse primer sequences used for each target gene synthesis in the RT-qPCR reaction.

Target Gene	Forward and reverse primer sequences
GAPDH	5'- CTGGAGAAACCTGCCAAGTATG -3'
	5'- GGTGGAAGAATGGGAGTTGCT -3'
Yap1	5'- GGCCATGCTCTCCCAACTGAA -3'
	5'- GGTTCATGGCAAAACGAGGGT -3'
Lats1	5'- GGGCGTGTACTTGTGGTCAT -3'
	5'- AGAAGTTTGCCAGTTGATAACCT -3'
Lats2	5'- CATTGCTCCTGAGGTGCTTCT -3'
	5'- GTGTGCCTTGAGGTCATCTGC -3'
